# Dynamics of Fertility-Related Traits in Tomato Landraces under Mild and Severe Heat Stress

**DOI:** 10.3390/plants11070881

**Published:** 2022-03-25

**Authors:** Barbara Farinon, Maurizio E. Picarella, Andrea Mazzucato

**Affiliations:** Department of Agriculture and Forest Sciences, University of Tuscia, 01100 Viterbo, Italy; b.farinon@unitus.it (B.F.); picarella@unitus.it (M.E.P.)

**Keywords:** fruit set, heat stress, *Solanum lycopersicum* L., stigma position, tomato

## Abstract

Studies on the reproductive dynamics under heat stress are crucial to breed more tolerant cultivars. In tomato, cultivars, breeding lines, and wild species have been evaluated for their response to heat stress. Here, we addressed the study to a panel of selected landraces representing traditional genotypes that usually show high adaptation to local environments. In two experiments, spaced by 12 years, we set-up an identical experimental design with plants transplanted at two different dates to expose the second field to thermic stress with natural fluctuations. Such a strategy resulted in both a mild and severe stress in the two years. The landraces showed wide variation for both vegetative and reproductive traits; all traits were affected by heat, mostly with a significant Genotype*Environment interaction. A high broad-sense heritability was estimated for plant height, stigma position, pollen viability, and fruit weight. Low heritability estimates were found for the number of flowers, fruit set, and yield. Despite the interaction, traits recorded under control and heat conditions were positively correlated. Multivariate analysis located the genotypes in a topography that was stable under all conditions, except under the harshest temperatures. The study revealed that landraces present a wide variability for the response of reproductive traits to thermic challenges and that such a variation could be useful to dissect the traits with higher heritability and identify quantitative trait loci for breeding more resilient varieties.

## 1. Introduction

Due to their sessile nature, plants are highly vulnerable to harsh environmental conditions such as increasing temperatures. It is thought that the global scenario of planet warming may have significant consequences on the life of plants with serious implications in agricultural crops [1].

Tomato (*Solanum lycopersicum* L.) is one of the world’s most important vegetable crops. Due to the high value of its fruit in terms of versatility, making it suitable for fresh consumption and for numerous types of processed products, tomato is an important dietary source of nutritional and health-related compounds [2]. Although tomato plants can grow under a wide range of temperatures, optimal reproduction and fruit set are limited when the day’s maximum exceeds 32 °C and the night’s minimum falls below 21 °C [3]. High temperature impairs plant growth at both the vegetative and the reproductive level [4,5]. At the vegetative level, photosynthesis is very sensitive to high-temperature stress, and heat is one of the most important causes of dry matter reduction in many crops [6,7]. Estimates range up to a 17% decrease in crop yield for each degree Celsius increase in average growing season temperature [8].

In many vegetables, such as tomato, reproductive development is more sensitive to and therefore more vulnerable to heat than the vegetative growth [9,10]. Heat effects indicate that in tomato, the male function is generally more exposed to damage than the female one [3,11,12]. The most sensitive stage is at early stamen development, when the anther has entered meiosis, which occurs about ten days before anthesis. Later, heat stress impairs tapetum functionality, leading to the inhibition of microgametogenesis [13]. Heat also disturbs pollen release, pollen transfer to the stigma surface, pollen tube growth, and finally fertilization [14]. All these effects result in reduced pollen grain number and viability [15] and ultimately in poor fruit set [16,17].

An indirect effect of heat stress on tomato fertility is the reaction of pistil growth, which, in certain genotypes, may push the style outside the anther cone, leading the stigma to an exserted position [18,19,20]. Whether exserted stigmas are common in wild tomato relatives, domestication caused the pyramiding of mutations, contributing to the progressive insertion of the stigma within the antheridial cone, thus ensuring strict autogamy, optimal pollination, and fertility [21]. The molecular basis for the control stigma position (SP) has started to be elucidated; a major quantitative trait locus (QTL) was positioned on the long arm of Chr2 [22] and the underlying gene, later identified as encoding a transcription factor that regulates cell elongation in developing styles [23]. Other genes involved in SP control have recently been suggested by transcriptional analyses [20,24] or by validation of candidates [25,26]. In cultivated tomato, a tendency toward an exserted SP is retained by many cultivated genotypes, especially landraces and primitive cultivars [27,28]; frequently, an increase in stigma exsertion is observable in these genotypes under heat stress [17,18].

Analyses of reproductive thermotolerance has been carried out in several tomato genotypes, including cultivars [11,29,30,31,32,33], breeding lines [19,32,34] and wild relatives [7,35]. Up to date, few studies have addressed the response to heat in tomato landraces [36,37], those cultivated populations adapted to specific climatic and agronomic conditions and preserved by farmers [38]. Opposite to modern breeding material that has been selected for high fertility (style insertion, high number of flowers, good fruit setting), landraces represent more ‘primitive’ genotypes that, beside possible defects in disease resistance and productivity, combine a high environmental adaptation and fruit quality. These traits raise great interest for both the valorization of traditional tomatoes and the use of ‘traditional’ genes in breeding programs. Landraces also represent a valuable experimental material to understand the mechanisms that underlie the variation in fertility in response to heat [39] or other abiotic [40] challenges.

In this work, we studied the phenotypic response to high temperatures in seven tomato Italian landraces and in three cultivars, diversified for origin, growth habit, SP, and fruit traits. Two parallel trials were repeated in a 12-year life span, subjecting the plants to mild heat stress in the first and to harsher temperatures in the second. This information will be a useful basis to explore the mechanisms affecting fertility under optimal and suboptimal climatic conditions and finally help in harnessing sustainability with the breeding of more resilient tomato cultivars.

## 2. Results

### 2.1. Selected Genotypes and Thermic Regimes in the Experimental Fields

Ten selected tomato genotypes were grown in two years in an open field. Landraces included genotypes with different fruit shapes and use: a round type with dual aptitude (TPer), flattened-ribbed types for fresh consumption (SBol, Spa, CFio), an elongate (SMar), a pear-shaped (Can), and an oxheart (CBue) type (Table 1). Control cultivars were vintage varieties, including two processing (CHI and SAL) and a fresh market (MON) type. Three landraces (SBol, Spa, and TPer) originated close to the environments used for the experiments and could be considered autochthonous, whereas the others were originated in more distinct environments.

Except for the two processing cultivars, all genotypes had an indeterminate growth habit (Table 1). As determined in previous studies [27], fruit weight (FW) ranged from 50–60 (SMar) to 140-150 (TPer) g and the value of SP from completely inserted to very exserted (Table 1).

The years of cultivation were 2007 and 2019; in both experiments, two transplanting dates, spaced by one month, were adopted in order to subject fruit setting of the first trusses to optimal conditions with the early transplant (hereafter referred to as control fields) and to heat stress with the second (referred to as heat fields). Such a strategy resulted in a difference of the average minimum, mean, and maximum temperatures experienced by the plants from the transplant to the blossom of the second truss of about 2–4 °C (Table 2). In addition, whereas data of average daily temperature variation were similar in the control fields in the two years, those registered in the heat field were much higher in 2019 compared to 2007. In 2007, half of the 40 d of monitored temperature in the heat field had a Tmax above 30 °C, whereas this occurred only seven times in the control field; in 2019, these values were 90% and 50%, respectively. Considering all hourly measurements (n = 960), in 2007, about 14% were above 30 °C in the heat and less than 3% in the control field; the same parameter was 26% and 13%, respectively, in 2019 (Table 2). These records indicated that plant growth was subjected to different thermic regimens in the two cultivations and in both years. In 2007, the second field experienced continuous mild heat, whereas in 2019, it experienced a more severe thermic stress.

### 2.2. Effect of Heat Stress on Vegetative and Reproductive Traits

To meet, or approach, the requirement of homoscedasticity, several variables were subjected to transformations for parametric analyses (see Materials and Methods). When all three main factors Code, Year, and Field were included in the analysis of variance (ANOVA), plant height (PH), SP, and pollen viability (PV) showed high significant second- and third-order interactions; the number of flowers per inflorescence (NF) and fruit set (FSET) showed a lower level of interaction and only for some factor combinations (Table S1). When a separate analysis was carried out for the two years, SP and PV showed a significant Code*Field interaction in 2007; in 2019, all variables except PV and Number of seeds per fruit (SxF) showed significant interaction (Table 3). One-way ANOVA revealed significant differences for all variables under all conditions, with the only exception of yield (Y) in the heat field (Table S2).

**Figure 1:**
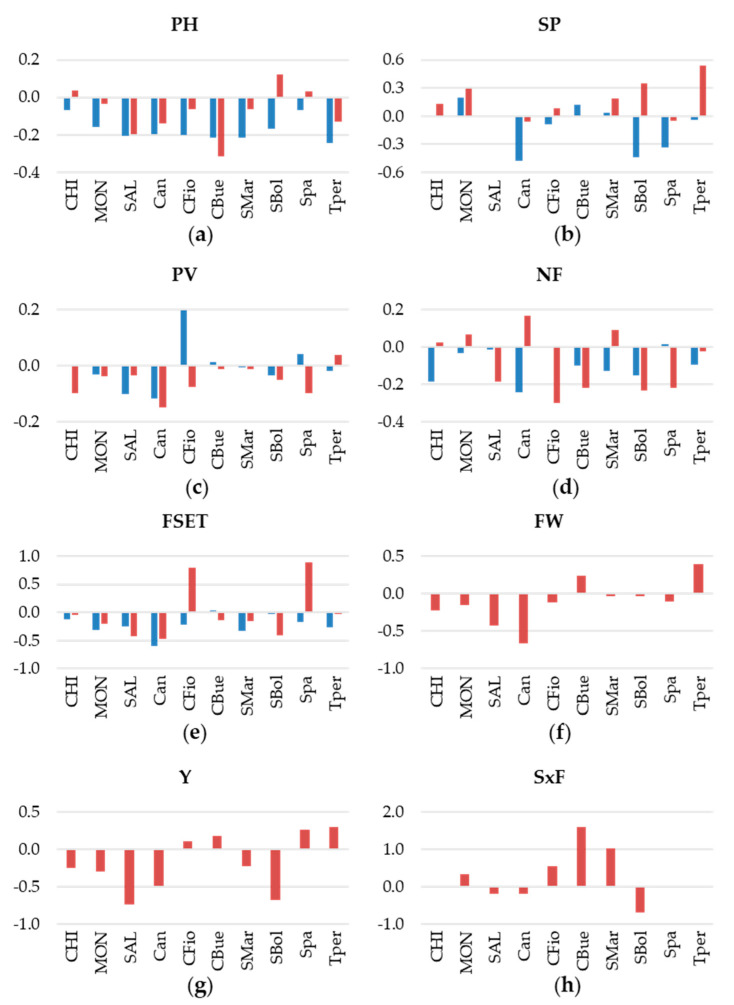
Relative variation in the heat versus the control field in 2007 (blue bars) and in 2019 (red bars) for the measured variables: (**a**) plant height (PH), (**b**) stigma position (SP), (**c**) pollen viability (PV), (**d**) number of flowers per inflorescence (NF), (**e**) fruit set (FSET), and, only in 2019, for (**f**) fruit weight (FW), (**g**) yield (Y), and (**h**) number of seeds per fruit (SxF). Variety names are abbreviated as follows: Chico III, CHI; Monalbo, MON; Saladette, SAL; Canestrino di Pisa, Can; Costoluto fiorentino, CFio; Cuor di bue, CBue; San Marzano, SMar; Scatolone di Bolsena, SBol; Spagnoletta, Spa; Tondo Perugia, Tper.

CFio, CBue, Can, and MON were the tallest varieties in 2007 in both fields; however, PH in the heat was generally lower than in the control field (Figure 1a; Table S3). Plants in 2019 were generally smaller than in 2007; however, they also showed a PH decrease under heat conditions (Figure 1a).

SP ranged from completely inserted under all conditions, such as in CHI and SAL, to highly exserted, as in CFio, CBue, and SMar (Table S3). In 2007, no genotype showed a consistent SP increase comparing control and heat fields; by contrast, in 2019, MON, SBol, and TPer showed a remarkable extrusion of the stigma under heat (Figure 1b).

PV under control conditions ranged from 100% (SBol) to 81.4% (CFio) in 2007 and from 96.6% (MON) to 80.2% (SMar) in 2019 (Table S3). Comparing the heat with the control field, several genotypes showed a decrease in PV, which was, however, year-specific; only Can showed a substantial reduction in PV in both experiments (Figure 1c).

As with PH, NF was strongly affected by heat conditions in many genotypes; those with substantial negative ΔNF in both years were CBue and SBol, although the highest reductions were found in Can in 2007 (−24%) and in CFio in 2019 (−30%; Figure 1d, Table S3).

Under control conditions, FSET was higher in cultivars; SAL had the highest value in both 2007 (about 80%) and 2019 (68%). The lowest FSET values were scored in Can and CBue, the genotypes having obovoid and heart-shaped big fruits (Table S3). Under heat, the lowest setting was still registered for Can and CBue; in addition, the cultivars MON and SAL had decreased FSET under both conditions (Figure 1e).

FW, Y, and SxF were only measured in 2019. Decreases in FW under heat were recorded in six genotypes, with SAL and Can showing the highest deltas (Figure 1f). This had a consequence in estimated Y, where these varieties had the highest decreases together with SBol (Figure 1g). SAL, Can, and SBol also showed a lower average SxF in the heat field; intriguingly, few genotypes, such as CBue and SMar, showed an increase in SxF under stress (Figure 1h).

### 2.3. Estimates of Heritability under Control and Heat Field Conditions

In 2007, high values of broad-sense heritability (h^2^_B_) were found for PH, SP, and PV, but not for NF and FSET (Table 4). In 2019, the highest h^2^_B_ values were found for the same traits, with the addition of FW. In 2007, values estimated under heat conditions were lower than those estimated in control fields, except for NF that showed no variation. In 2019, heritability in the heat field was lower than in the control one for PH, FSET, and Y, but it was the same or higher for the remaining variables (Table 4).

### 2.4. Correlations among Traits

**Figure 2:**
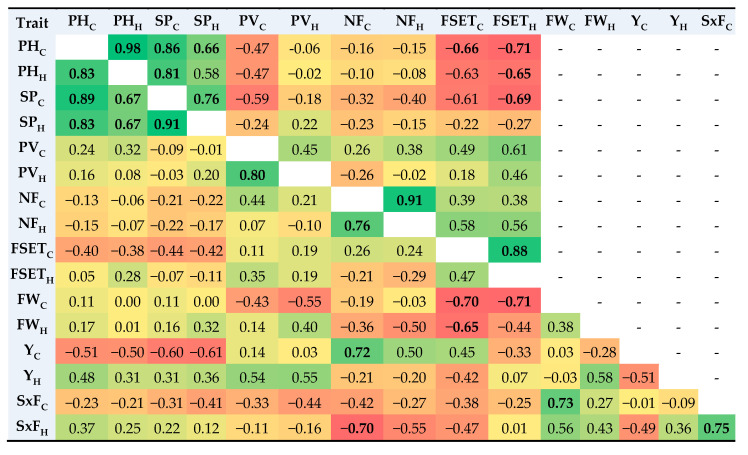
Pearson’s correlation coefficients among measured traits (plant height, PH; stigma position, SP; pollen viability, PV; number of flowers per inflorescence, NF; fruit set, FSET; fruit weight, FW; yield, Y; number of seeds per fruit, SxF) in 2007 (upper triangle) and in 2019 (bottom triangle). Subscripts “C” and “H” indicate values detected in the control and heat field, respectively. Coefficients are reported in cells with green to red color, ranging from high positive to high negative correlations; coefficients statistically significant for *p* ≤ 0.05 are written in bold.

All measured traits except PV showed highly significant positive correlations between means recorded under control and heat conditions in 2007 (Figure 2, upper triangle). In addition, PH in the control field showed correlations with SP under both conditions. Both FSET values were negatively correlated with PH, although FSET in the control failed to reach a significant correlation with PH in the heat field (r = −0.63, *p* = 0.052). FSET under heat conditions was negatively correlated with SP under control conditions.

In 2019, all traits showed significant positive correlations between means recorded under the control and heat conditions, with the exceptions of FSET, FW, and Y (Figure 2, lower triangle). Both PH measures were correlated with SP values. NF in the control field was positively correlated with Y in the control and negatively with SxF in the heat field. FSET in the control field was negatively correlated with FW under both conditions measured. Finally, under control conditions, bigger fruits also had more seeds (Figure 2).

**Figure 3:**
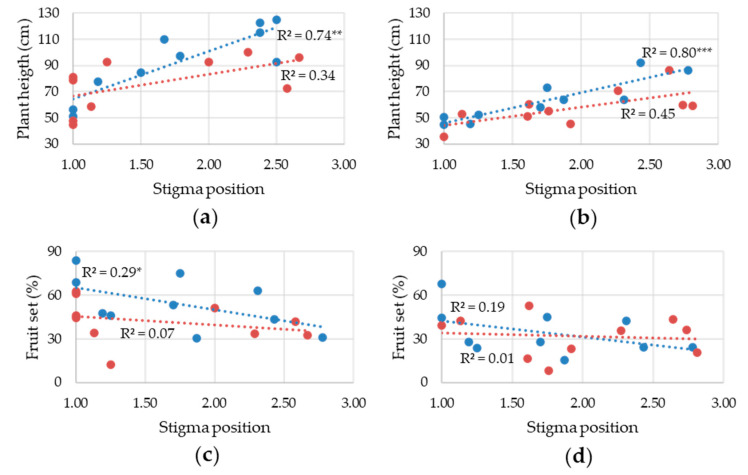
Linear regression between stigma position and plant height in (**a**) 2007 and (**b**) 2019 and between stigma position and fruit set in (**c**) 2007 and (**d**) 2019. In each graph, the regression in the control (blue dots and line) and in the heat (red dots and line) field is reported; for each regression, the determination coefficient (R^2^) and the significance of the regression analysis of variance are reported. *, **, and *** indicate significant regressions for *p* ≤ 0.05, 0.01, and 0.001, respectively.

Correlations of SP with PH and FSET were further studied by regression analysis. PH and SP confirmed a direct relationship that was significant in both years but only under control conditions (Figure 3a,b). The relationship between SP and FSET showed a negative trend under control conditions, which was significant only in 2007; in the heat field, such a relationship was lost (Figure 3c,d).

**Figure 4:**
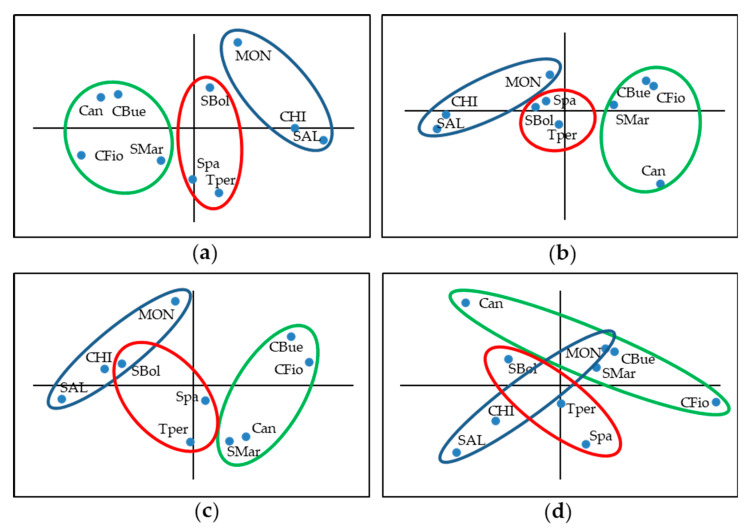
PC1 × PC2 plots of the ten studied varieties after principal component analysis of plant height, stigma position, pollen viability, number of flowers per inflorescence, and fruit set according to the cultivation under (**a**) control and (**b**) heat conditions in 2007 and under (**c**) control and (**d**) heat conditions in 2019. Variety names are abbreviated as follows: Chico III, CHI; Monalbo, MON; Saladette, SAL; Canestrino di Pisa, Can; Costoluto fiorentino, CFio; Cuor di bue, CBue; San Marzano, SMar; Scatolone di Bolsena, SBol; Spagnoletta, Spa; Tondo Perugia, Tper.

Principal component analysis was separately carried out for the two years and the two fields. In 2007, the first two principal components (PCs) explained more than 78% and 70% of the total variance. In the control field, the first component was negatively loaded by PH and SP and positively by FSET; in the heat field, the loading factors were the same although with different signs (Table S3). PC2 was loaded by NF in the control and by PV under heat conditions. Genotype coordinates separated the varieties along the first axis, with cultivars showing the highest PC1 values under control conditions (Figure 4a). Among the studied landraces, those with the origin in the Latium and Umbria region, which can be considered autochthonous (SBol, Spa, TPer), still had positive PC1 values, corresponding to smaller plants, inserted stigma, and higher fertility, whereas the remainder were located on the negative part of the axis (Figure 4a). The heat conditions experienced in this year did not change the variety distribution, which was specular but parallel to that detected in the control field (Figure 4b).

Multivariate analysis on 2019 data explained 76% and 63% of the variability with the first two PCs under control and heat conditions, respectively. In this case, PC1 was positively loaded by PH and SP under both conditions (Table S3). In the control field, FSET also had an important negative loading on PC1. Main loadings for PC2 were PV (positive) in the control and FSET (negative) in the heat field. PC1 loadings made the topography of accessions under control conditions mirror the one observed in 2007; the general distribution of genotypes was similar except for SBol that showed PC1 values comparable to the cultivars (Figure 4c). Under heat conditions, the topography was changed, with the cultivar MON located between landraces and the non-autochthonous landrace Can positioned among the cultivars CHI and SAL, but with a different PC2 score due to a lower FSET (Figure 4d). Notably, autochthonous landraces also maintained an intermediate position under these harshest tested conditions (Figure 4d).

## 3. Discussion

Due to the ongoing climatic changes and temperature increase, studies on reproductive dynamics in crops challenged by heat stress are crucial to gain knowledge into tolerance mechanisms and insights to breed more tolerant cultivars. Structures ensuring tight temperature control, such as growth chambers and phytotrons, are usually adopted for heat stress studies. However, controlled environments suffer from space limitations and, usually, supply heat stress in continuous regimes without those fluctuations that occur in the open field. In our work, the heat-challenging environment was achieved by postponing the transplant to delay the flowering season in the hottest days of the year. This strategy was adopted to maintain growth conditions more similar to the reality and that could more successfully be faced by stressed plants. Mild heat conditions continuously subjected for prolonged periods in fact caused much more severe effects than those recorded in our mild heat conditions in 2007 [31,33,35]. In addition, experiments repeated in time usually consider consecutive years. By contrast, we tested two different growing seasons spaced by a 12-year time lapse, which experienced a warmer season that was mild in 2007 and harsh in 2019.

Under almost all conditions, the studied genotypes showed a negative ΔPH, proving that plants experienced physiological stress in the heat fields. This response has been previously reported in cultivated tomato and wild relatives and associated with disturbance of the metabolite supply and transport pathways in specific organs at specific developmental stages [7,17,19]. Negative effects on growth were also reported under drought stress [40].

During flower development, differences in SP between genotypes and a significant Genotype*Environment (G*E) interaction have been reported in previous studies in both cultivated and wild genotypes [35,41]. In our experiments, the two cultivars CHI and SAL maintained an inserted style under heat, confirming thermotolerance for this trait [29,35,42,43]. Landraces with an inserted stigma (SBol, Spa, and TPer) did not change phenotype under mild heat in 2007; however, under harsh heat conditions in 2019, SBol and TPer increased their SP, thus showing G*E interaction for this trait. In parallel, two genotypes with exserted stigma, CFio and CBue, showed a stable expressivity of the trait. In 2007, three genotypes had a stigma more inserted under heat than under control conditions. Thus, the reaction of landraces for SP under mild or severe heat stress is very variable.

The present study confirmed that PV in tomato shows genotypic diversity [31] and may be negatively affected by heat stress [17,33]. Although CHI and SAL showed a negative ΔPV, all the three cultivars maintained good PV under heat conditions. The most sensitive genotype for PV was Can, a landrace with pear-shaped fruits from Tuscany, showing negative ΔPV in both years.

NF was negatively affected by heat in both years in several genotypes; this confirmed that temperature affects this trait in tomato [31,33,41]. Under mild heat conditions, G*E was not significant for NF, as in other studies [33], but under less favorable conditions, a significant interaction was observed. Thus, increasing the severity of stress conditions, the genotypic difference in resilience for this trait is highlighted.

FSET in our experiment was estimated after open pollination; as reported in other studies [17,19,44], it decreased with temperature, but with a strong G*E interaction. Although the reportedly thermotolerant cultivars CHI and SAL showed a negative impact of heat on FSET, they maintained the highest setting under heat conditions due to the good ability already expressed under control conditions. The highest negative ΔFSET among landraces was registered in Can in both years, which could be related to a decreased PV. Interestingly, two landraces, Spa and CFio, showed increased FSET in 2019; this estimation was correlated to a good resilience of SP in these genotypes.

In 2007, PH, SP, and PV showed high heritability estimates. High h^2^_B_ values for SP agree with previous observations [30,45]. However, the h^2^ value calculated under heat was about 10% lower than that estimated under control conditions. These data suggest that a favorable SP can be harnessed by breeding, although few QTLs for this trait are known. For many years, the only known gene controlling SP was *Se2.1* [23], but recently, other positions have been identified [46] together with new candidate genes [12,25]. The SP variability present in landraces may be exploited to unravel new QTLs for this trait by biparental or linkage disequilibrium-based QTL mapping.

Similarly, heritability estimates for PV were high in 2007 and similar to those reported in other studies [35]. Although high PV h^2^_B_ estimates were not confirmed in 2019, also for this trait, QTL discovery and marker-assisted selection are plausible perspectives.

A low estimate of h^2^_B_ was found for NF in both years, as already seen in tomato [47] and in other *Solanaceae* [48]. The same was found for FSET; values ranging from 0.21 to 0.31 were reported in heat-tolerant tomato lines [16] and of about 0.40 in other materials [33]. These evaluations explain why markers for FSET have hardly been reported [49] and suggest that it would be necessary to dissect FSET into single components to better address yield stability in heat-challenged tomatoes. The drastic fall in heritability for Y under the harshest conditions of the 2019 heat field supported this conclusion.

In 2007, except for PV, means recorded under control and heat conditions were positively correlated, suggesting that the interactions estimated by ANOVA were due to the behavior of few genotypes. PH was correlated with SP, in part because the two cultivars with a stably inserted stigma also had determinate growth. However, landraces with an indeterminate habit and inserted stigma also had a low PH, suggesting a link between moderate vegetative growth and stigma insertion. As reported in other studies, we detected a negative correlation between SP and FSET, but a significant regression was only found under control conditions in 2007. Thus, FSET variation may be related to SP, but not to the specific SP increase that may occur under heat stress. The relative importance of SP in relation to FSET has been controversial; not always the highest levels of exertion corresponded to the lowest fruit set [31,42], indicating that exserted SP is not always a major restriction to FSET. Here, we lacked the detection of the correlation between PV and FSET that was previously reported [30,32,33,50].

In 2019, all traits showed significant positive correlations between means recorded under control and heat conditions, except for FSET, FW, and Y. This is again an indication that productive traits are more affected by a stressing environment.

The multivariate analysis of the traits recorded in different environments showed a stable behavior of cultivars with determinate growth, showing low PH and SP and high FSET. MON followed a distinct trend, having taller plants and SP not completely inserted. The landraces Can, CBue, CFio, and SMar, which were classified as non-autochthonous, had opposite topography in the graph due to the taller plants and lower values for fertility traits. Interestingly, landraces classified as autochthonous, such as SBol, Spa, and TPer, had an intermediate position, which was very stable in mild heat. However, these genotypes also showed a high variation of the measured traits and decrease in fertility under the harshest tested conditions.

## 4. Materials and Methods

### 4.1. Plant Material and Growth Conditions

Seven Italian tomato landraces were selected based on a previous characterization [27] to maximize the diversification for vegetative and reproductive traits (Table 1). Two of them (SBol and Spa) originated in the Latium region, which is also where the experiments were carried out and are listed in the Voluntary Regional Register issued by the regional Law 1/03/2000–N.15 “Protection of autochthonous genetic resources of agricultural interest” (https://www.arsial.it/biodiversita/registro-volontario-regionale/, accessed 21 January 2022). Together with TPer, these landraces are referred to as autochthonous, in contrast with the remainder, which originated in more distant environments. Three vintage cultivars were selected as references among those frequently adopted by the scientific community; these included two processing (Chico III and Saladette) and a fresh market (Monalbo) true breeding line. All the seed stocks were available from the collection held by the authors at the University of Tuscia.

The ten genotypes were used in two field trials, the first established in 2007 at the Experimental Farm of the University of Tuscia at Viterbo, Italy (42°25′07′′ N, 12°06′34′′ E) and the second in 2019 at a farm located in Montefiascone (42°32′25′′ N, 12°2′13′′ E). The two sites share very similar climatic regimes. In both fields, to expose plant flowering and fruit setting to different temperature regimes, the experimental design was replicated by adopting an early (1st of June, control field) and a late (1st of July, heat field) date of transplant in an open field. In each year and field, ten plants per accession were arranged in twin rows and grown with the same agronomic techniques and inputs, corresponding to the standard agronomic practices for fresh market tomatoes. All genotypes, including the cultivars with a determinate growth habit, were grown on tutors with a single shoot. Lateral shoots were weekly removed, and plants were left to open pollination.

During both experiments, the temperature was recorded on an hourly basis for the first 40 d after the transplant; this time span corresponded to the flowering and setting of the first two trusses in most genotypes. For the period of temperature measurements, the average daily minimum (Tmin), maximum (Tmax), and mean (Tmean) temperature were calculated, together with the daily variation (Tdiff = Tmax − Tmin). In addition, the number of days (n = 40) and measurements (n = 960, expressed in %) with temperatures below 15 °C and above 30 °C were counted or calculated, respectively.

### 4.2. Phenotyping of Vegetative and Reproductive Traits in the Control and Heat Fields

In the four experiments, phenotypic data were collected following the same protocol. Plant height (PH) was measured 40 d after transplanting. In the same date, approximately corresponding to the setting of the second truss, the position of the stigma with respect to the anther cone (SP) was recorded on each plant by scoring all the open flowers (1, inserted; 2, at the level of stamens; 3, exserted; 4, very exserted).

At the same date, pollen viability was estimated on four flowers at the anthesis sampled from two plants of each accession; anthers were dissected, and the pollen released with a razor was observed by light microscopy with an Axioskop 2 FS plus (Zeiss, Jena, Germany) after staining with two drops of a 1% (*w/v*) solution of orcein (Sigma-Aldrich, St. Louis, MO, USA) in glacial acetic acid. A minimum of 100 pollen grains per slide were counted and classified as viable or nonviable based on their stainability and morphology. Pollen viability (PV) was expressed as the ratio of the stainable pollen grains over the total number of specimens observed in percent.

Each year, on the 20th of August and September for the two fields, when ripe fruits were present, the number of flowers per inflorescence (NF) and the number of fruits set in the first three trusses were counted. Fruit set (FSET) was calculated as the ratio between the number of fruits over the total number of flowers in each observed truss and expressed as percent.

In the 2019 trial only, on a single plant basis, at harvest, all red fruits were weighed, and the mean fruit weight (FW) was calculated. The NF counted on the first three trusses and FW were used to calculate a potential yield estimation (Y). From the red fruit sample, seeds were extracted, counted, and referred to as mean number of seeds per fruit (SxF).

### 4.3. Statistical Analysis

To meet, or approach, the homoscedasticity requirement, variables SP and FW were subjected to logarithmic transformation, PV and FSET in arcsin, and NF and SxF in square root. All parametric analyses were conducted with transformed variables, although means were reported in the original measurement units. Analysis of variance (ANOVA) was carried out following the general linear model procedure implemented in the PROC GLM of SAS [51]. For the variables NF and FSET, a preliminary analysis was carried out according to a four-factor factorial design, with “Code,” “Year,” “Field,” and “Truss” as main factors. As the third-order interaction was not significant in this analysis, data have been mediated over the three trusses and subsequent analyses carried out with lower-complexity models. Separate analyses for each different year and field were carried out when interaction effects were significant. The mean separation was obtained after Duncan’s multiple range test.

Once mean values for each year and field had been obtained, the variation between the two growing conditions within the same year was determined as the percent variation of the heat field compared to the control field according to the formula ((ValueH − ValueC)/ValueC) × 100.

Broad-sense heritability (h^2^_B_) was calculated as σ^2^_gen_/σ^2^_tot_ using variance estimates derived from one-way ANOVA. σ^2^_gen_ was calculated as (σ^2^_between_ − σ^2^_within_)/k, where k was the number of replicates for each observation. σ^2^_within_ was considered an estimate of σ^2^_e_. σ^2^_tot_ was σ^2^_gen_ + σ^2^_e_.

Pearson’s correlation coefficients (r) among the variables estimated in each field were separately calculated for each year of experimentation, using PROC CORR and PROC REG implemented in SAS [51]. Using the five variables measured in all fields (PH, SP, PV, NF, and FSET), a principal component analysis was carried out for each year and field by using the PROC PRINCOMP procedure [51].

## 5. Conclusions

Altogether, the results support the view that low fruit set under high temperatures is not the consequence of a single sensitive factor, but instead of a simultaneously impaired complex of components. The dissection of the effect of each trait involved allowed estimating those aspects with higher heritability, such as SP and PV, that may represent reliable breeding targets for a better reproductive performance under harsh conditions. Landraces show wide variability for vegetative and reproductive phenotypes related to heat tolerance. A higher, and wider, characterization of this material will help elucidate genetic mechanisms underlying tolerance and identify genes and alleles that are involved. This knowledge will be the basis for assisted breeding programs targeted to select new tomato varieties more resilient to the environmental changes.

## Figures and Tables

**Table 1:** Name, abbreviation, origin, and previous characterization of the ten studied genotypes, including growth habit (GH; D determinate, I, indeterminate), fruit shape (FS), fruit weight (FW, g), and stigma position (SP, score, 1 = inserted; 2 = as the same level as stamens; 3 = slightly exserted; 4 = very exserted).

Type	Name	Abbreviation	Origin	Previous Characterization ^1^			
				GH	FS	FW	SP
Cultivar	Chico III	CHI	- ^2^	D	Block-shaped	70–80	1.0
	Monalbo	MON	-	I	Round	60–70	1.5
	Saladette	SAL	-	D	Plum-shaped	70–80	1.0 ^3^
Landrace	Canestrino di Pisa	Can	Tuscany	I	Pear-shaped	130–140	1.3
	Costoluto fiorentino	CFio	Tuscany	I	Flattened-ribbed	90–100	1.5
	Cuor di bue	CBue	Market	I	Heart-shaped	130–140	2.5
	San Marzano	SMar	Campania	I	Elongate	50–60	1.1
	Scatolone di Bolsena	SBol	Latium	I	Flattened-ribbed	130–140	1.0
	Spagnoletta	Spa	Latium	I	Flattened-ribbed	90–100	1.0
	Tondo Perugia	TPer	Umbria	I	Round	140–150	1.0

^1^ Ref. [27]. ^2^ not applicable. ^3^ data from unpublished observations.

**Table 2:** Dynamics of minimum (Tmin), mean (Tmean), and maximum (Tmax) daily temperatures and daily variation (Tdiff) in the 40-d period from transplanting to flowering of the 2nd truss in the control and heat experimental fields and number of days (n = 40) and records (n = 960, reported in %) below 15 °C and above 30 °C in the same period in the two years. For Tmin, Tmean, Tmax, and Tdiff, the range is reported in brackets.

Variable	2007		2019	
	Control	Heat	Control	Heat
Tmin (°C)	14.2 (10.1–20.4)	15.7 (10.8–20.5)	15.3 (9.4–22.6)	18.4 (14.9–22.6)
Tmean (°C)	20.9 (15.1–26.6)	23.4 (18.5–27.2)	22.7 (14.7–17.5)	26.4 (20.3–30.3)
Tmax (°C)	27.8 (19.1–33.7)	30.9 (24.1–35.6)	28.3 (17.5–36.6)	32.8 (21.4–36.9)
Tdiff (°C)	13.6 (6.7–17.9)	7.4 (5.5–8.9)	13.0 (5.1–17.0)	14.5 (4.0–19.0)
No. of days with Tmin ≤ 15 °C	28	15	16	1
No. of days with Tmax ≥ 30 °C	7	20	20	36
No. of records with T ≤ 15 °C (%)	11.8	6.3	18.1	0.001
No. of records with T ≥ 30 °C (%)	2.7	13.9	13.2	26.0

**Table 3:** Two-way factorial analysis of variance for plant height (PH), stigma position (SP), pollen viability (PV), number of flowers per inflorescence (NF), and fruit set (FSET) in 2007 and 2019, and for fruit weight (FW), yield (Y), and number of seeds per fruit (SxF) in 2019. *, **, and *** indicate significance of the main factors, Code and Field, and of their interaction for *p* ≤ 0.05, 0.01, and 0.001 respectively.

Trait	2007			2019		
	Code	Field	Code × Field	Code	Field	Code × Field
PH	***	***	-	***	***	***
SP	***	***	***	***	***	**
PV	***	-	***	**	***	-
NF	***	**	-	***	***	**
FSET	***	***	-	***	*	***
FW				***	*	**
Y				-	***	***
SxF				***	-	-

**Table 4:** Values of broad-sense heritability estimated in control and heat experimental fields for plant height (PH), stigma position (SP), pollen viability (PV), number of flowers per inflorescence (NF), fruit set (FSET) in 2007 and 2019 and, only in 2019, for fruit weight (FW), yield (Y), and number of seeds per fruit (SxF).

Trait	2007		2019	
	Control	Heat	Control	Heat
PH	0.94	0.85	0.81	0.75
SP	0.84	0.74	0.77	0.76
PV	0.84	0.77	0.32	0.51
NF	0.27	0.28	0.23	0.34
FSET	0.33	0.29	0.33	0.29
FW	- ^1^	-	0.62	0.74
Y	-	-	0.37	0.13
SxF	-	-	0.51	0.56

^1^ not available.

## Data Availability

The data presented in this study are available in the article and Supplementary Materials.

## Supplementary Materials

The following supporting information can be downloaded at: https://www.mdpi.com/article/10.3390/plants11070881/s1, Table S1: Three-way factorial analysis of variance for plant height (PH), stigma position (SP), pollen viability (PV), number of flowers per inflorescence (NF), and fruit set (FSET). Significance of the main factors “Code,” “Year,” and “Field” and of their first- and second-order interactions. *, **, and *** indicate significance for *p* ≤ 0.05, 0.01, and 0.001, respectively; Table S2: One-way analysis of variance for plant height (PH), stigma position (SP), pollen viability (PV), number of flowers per inflorescence (NF), and fruit set (FSET) recorded under control and heat conditions in 2007 and 2019, and for fruit weight (FW), yield (Y), and number of seeds per fruit (SxF) under control and heat conditions in 2019. For each variable, the values under control and heat conditions are indicated with “C” and “H” subscripts, respectively. Means followed by the same letter are not statistically different for *p* ≤ 0.05 after Duncan’s multiple range test. The percent variation between the two growing conditions is reported and indicated as Δ variables. Table S3: Percentage of explained variation, trait loading values and coordinates of the ten studied varieties, for the two first Principal Components (PC) derived from five phenotypic variables (plant height, PH; stigma position, SP; pollen viability, PV; number of flowers per inflorescence, NF; fruit set, FSET). Variety names are abbreviated as follows: Chico III, CHI; Monalbo, MON; Saladette, SAL; Cane-strino di Pisa, Can; Costoluto fiorentino, CFio; Cuor di bue, CBue; San Marzano, SMar; Scatolone di Bolsena, SBol; Spagnoletta, Spa; Tondo Perugia, Tper.
